# The Associations of Birthweight for Gestational Age Status with Its Differential 0–2 Year Growth Trajectory and Blood Pressure at Two Years of Age in Chinese Boys and Girls

**DOI:** 10.3390/nu15040979

**Published:** 2023-02-16

**Authors:** Fengxiu Ouyang, Xiaobin Wang, Jonathan C. Wells, Xia Wang, Lixiao Shen, Jun Zhang

**Affiliations:** 1 Ministry of Education and Shanghai Key Laboratory of Children’s Environmental Health, Xinhua Hospital, Shanghai Jiao Tong University School of Medicine, 1665 Kong Jiang Road, Shanghai 200092, China; 2 Center on the Early Life Origins of Disease, Department of Population, Family and Reproductive Health, Johns Hopkins University Bloomberg School of Public Health, Johns Hopkins University School of Medicine, Baltimore, MD 21205, USA; 3 Childhood Nutrition Research Centre, Population, Policy and Practice Research and Teaching Department, University College London, Great Ormond Street Institute of Child Health, London WC1N 1EH, UK

**Keywords:** birthweight to gestational age, large for gestational age (LGA), appropriate weight for gestational age (AGA), small for gestational age (SGA), growth trajectory, anthropometric measures, blood pressure

## Abstract

The first 1000 days of life represents a critical period for lifelong metabolic health. This study prospectively examined the contrasts between the growth trajectories of large, small, and appropriate sizes for gestational age (LGA, SGA, and AGA) term-born infants in their first two years, and their blood pressure at two years. In 2012–2013, 806 Chinese mother-newborn dyads were enrolled in the Shanghai Obesity and Allergy Birth Cohort Study. Repeated anthropometric measures were obtained at age 42 days, and at 3, 6, 9, 12, 18 and 24 months. Systolic and diastolic blood pressure (SBP, DBP) were measured at two years of age. Linear random effect models were employed to evaluate growth trajectory differences between LGA, SGA, and AGA infants. Of the study infants, 12.4% were LGA and 4.0% SGA. Length, weight, and weight-for-length z-score (ZWFL) were all consistently higher in LGA infants and lower in SGA infants than AGA infants. SGA infants had a higher ZWFL (0.11 unit/month; 95% CI: 0.04, 0.19) and a higher BMI (0.19; 95% CI: 0.09, 0.28 kg/m^2^ per month) growth velocity at age 0–6 months, relative to AGA infants. SGA was associated with 6.4 (0.4–12.4) mmHg higher SBP, and LGA was associated with 2.9 (95% CI −5.2, −0.5) mmHg lower DBP at two years of age in boys, however, not in girls. In conclusion, in this prospective birth cohort with repeated anthropometric measures and BP at two years of age, LGA, SGA, and AGA term-born infants manifested differential patterns of weight growth trajectory and BP, providing new insight into developmental origins of cardiometabolic health.

## 1. Introduction

The global obesity and type two diabetes epidemics affect all age groups, including fetuses and young children [1]. Obesity is a major risk factor for cardiovascular disease and diabetes in adults [2,3]. In China, the prevalence of child overweight and obesity (OWO) has increased dramatically in the past three decades from 1.8% in 1985 to 20.5% in 2014 [4,5]. Obesity at two years of age has been associated with a persistently higher risk of obesity in childhood and adulthood [6,7]. The first 1000 days of life represent a critical period for lifelong metabolic health, and for early interventions intended to reduce childhood adiposity, a known driver of cardiovascular diseases and diabetes in adult life [8].

Maternal nutritional status (prepregnancy body mass index (BMI) categories, gestational weight gain (GWG), and gestational diabetes mellitus (GDM)) also are associated with fetal growth and birthweight [9,10]. Women with obesity and excess GWG are commonly identified as being at increased risk for GDM and delivering infants who are large for their gestational age (LGA) [11,12]. Adverse health outcomes for infants born with low birthweight (or being small for their gestational age, SGA) have been key drivers of the developmental origins of health and disease (DOHaD) hypothesis [13,14]. In fact, both LGA and SGA in infancy have been linked to an increased risk of later cardiometabolic diseases [14]. Identification of infants at increased risk for obesity is the starting point for early interventions that could be adapted to individual risk status [15]. However, this requires a better understanding of the longitudinal growth patterns of LGA, AGA, and SGA infants, which may provide insights regarding their risk for obesity.

In this study, we aimed to examine the overall patterns of and contrasts in the longitudinal growth trajectories of term infants born as LGA, AGA, and SGA, taking into account different postnatal time periods across the first two years. We also examined the associations of birthweight for gestational age status with blood pressure (BP) at age two years in boys and girls. As infants born preterm have different growth trajectories [16], this study focused on term-born children.

## 2. Methods

### 2.1. Study Population

This report used data from the Shanghai Obesity and Allergy Birth Cohort Study. The baseline study of this cohort was conducted between June 2012 and February 2013 in Shanghai, China. Pregnant women were enrolled at two large tertiary hospitals. They were eligible to participate if they met the following criteria: (1) had a singleton pregnancy, (2) received routine prenatal care at the study hospitals, (3) planned to live in Shanghai for the next two years, and (4) were willing to participate in this study and provide consent. After obtaining written informed consent, trained study nurses conducted face-to-face interviews using a standardized questionnaire to collect general information including maternal prepregnancy weight, height, education, smoking and passive smoking during pregnancy.

After delivery, the study nurses reviewed hospital medical records using a standardized abstraction form to obtain maternal and infant clinical data, including maternal height, pregnancy complications (for example, GDM), the last weight before delivery, mode of delivery, infant sex, gestational age, and birthweight.

Postnatal infant follow-up investigations were conducted at age 6 months using a web-based questionnaire survey, followed by onsite visits at the ages of 1 year and 2 years to collect information on infant growth, feeding type (exclusive breastfeeding, mixed feeding, and formula), and passive smoking (yes/no). The two postnatal follow-up visits were conducted during June 2013–2014 (child aged 1 year) and June 2014 to April 2015 (child aged 2 years).

A total of 1243 women were eligible for the study and enrolled at baseline. Among them, 829 children had a postnatal follow-up visit. The 23 children born preterm (gestational age <37 weeks) were excluded. Thus, 806 children were included in the study.

All mothers provided signed informed consent The study was approved by the Institutional Ethical Review Committees of both Xinhua Hospital and the International Peace Maternity and Child Hospital.

### 2.2. Definitions of Birthweight for Gestational Age Status

The definitions of LGA, AGA, and SGA were based on Chinese sex-specific references for birthweight at each gestational week [17]. LGA was defined as birthweight for gestational age >90th percentile, AGA as the 10th–90th percentile, and SGA as <10th percentile in boys and girls, respectively. 

### 2.3. Definitions of Maternal Nutritional Status

The diagnosis of GDM followed the recommendation of the International Association of Diabetes and Pregnancy Study Groups (IADPSG), as described previously [18,19].

Based on the Chinese adult population BMI classification standards, maternal prepregnancy BMI (kg/m^2^) was classified into four categories: underweight < 18.5 kg/m^2^, normal weight 18.5–23.9 kg/m^2^, overweight 24–28 kg/m^2^, and obese ≥ 28 kg/m^2^ [20].

GWG was defined as the difference in maternal weight between prepregnancy weight and the weight before delivery [19]. GWG was classified into excessive, appropriate, and insufficient based on GWG above or below the recommended Institute of Medicine (IOM) 2009 guidelines according to prepregnancy BMI as follows: 12.5–18 kg (BMI < 18.5); 11.5–16 kg (BMI 18.5–24.9); 7–11.5 kg (BMI 25–29.9); and 5–9 kg (BMI ≥ 30) [21].

### 2.4. Child Anthropometric Measures and Infant Growth Trajectories

Repeated measures of child weight and length were obtained from postnatal childcare medical records at ages 42 days and 3, 6, 9, and 18 months, and onsite measures at ages 12- and 24 months. 

Length (in the supine position) and weight (using Seca 956 Scales) were routinely measured at postnatal healthcare visits by trained nurses at community healthcare centers in Shanghai, following the World Health Organization (WHO) protocol [22].

At postnatal 12- and 24-month follow-up visits, anthropometric measures, including weight, length, head circumference, skinfold thicknesses, and mid–upper arm circumference (MUAC) were measured by study nurses at the child follow-up center in our (tertiary) hospital. Weight was measured to the nearest 0.1 kg (Seca 956 Scale), length to the nearest 1 mm (Seca 416 Scale), and head circumference to the nearest 1 mm. Skinfold thicknesses were measured at three sites (triceps, subscapular and abdominal) to the nearest 0.5 mm by two study pediatricians using Lange calipers (Beta Technology, Santa Cruz, CA, USA).

Sex-specific z-scores were calculated for length for age, weight for length, and weight for age using WHO Child Growth Standards (WHO 2006) (http://www.who.int/childgrowth/standards/en/; accessed on 14 February 2023) [22]. 

### 2.5. Other Child Cardio-Metabolic Factors at Age 2 Years

At the postnatal 2 year follow-up morning visit, BP was measured by a study pediatrician and a blood draw was taken by study nurses. At least 30 min after the child arrived at our study center for the study visit, SBP and DBP were measured on the left arm with a mercury sphygmomanometer using an appropriate size cuff for arm circumference. Three readings were taken 1 min apart and the average was used in the analysis.

### 2.6. Data Analysis and Statistics

We compared maternal and child characteristics among the three groups of SGA, AGA, and LGA infants. Fisher’s exact test or χ^2^ tests were used to compare categorical variables and ANOVA F-tests for continuous variables. Spaghetti plots were used to illustrate the 6340 longitudinal anthropometric measures (length, weight, and ZWFL) from age 0–25 months, and a LOWESS regression plot was used to visualize AGA, SGA, and LGA status in 806 term-born children (418 boys and 388 girls).

Linear random effect models, with a random intercept and an exchangeable correlation structure to accommodate longitudinal repeated measures, were used to evaluate the differences in the growth velocity of anthropometric measures (y) with age in LGA (i.e., β3 + β4) and in SGA (i.e., β3 + β5) infants, compared to the mean growth trajectory with age in AGA (i.e., β3) infants. Compared to the growth slope (i.e., β3) for AGA infants, the growth trajectory difference was β4 for LGA infants (i.e., interaction effects, LGA * Age) and β5 for SGA infants (i.e., SGA * Age). Data were analyzed by age from 0-6 months and 7-25 months, separately. Specifically, for Model 1: anthropometric measures (y) = β0 + β1 * LGA + β2 * SGA + β3 * child age + β4 * child age * LGA + β5 * child age * SGA. For Model 2: Model 1 + covariates including maternal passive smoking during pregnancy (yes/no), mode of delivery, infant sex, child age, infant feeding type (exclusive breastfeeding, mixed feeding, and formula) at age 0–6 months, and child passive smoking (yes/no). The covariates included in the regression models were used to control for potential confounders and gain more precise estimates.

Furthermore, we used generalized estimating equation (GEE) linear regression to evaluate the associations between birthweight for gestational age status (LGA, SGA, and AGA) and infant length, weight, BMI and ZWFL at age 0–6 months and 7–25 months, respectively. The adjusted models included child age, child age^2^, maternal passive smoking during pregnancy (yes/no), mode of delivery, infant sex, feeding type (exclusive breastfeeding, mixed feeding, and formula) at age 0–6 months, and child passive smoking (yes/no).

Linear regressions were used to examine the association between birthweight for gestational age status (LGA, SGA and AGA) and SBP and DBP at age 2 years in boys and girls, separately, with adjustment for child age and ZWFL at age 2 years.

All analyses were conducted using SAS 9.2 software (SAS Institute, Cary, North Carolina) and STATA 15.1 (Corp, College Station, TX, USA).

## 3. Results

This study included 806 mothers and their term-born children. Mean maternal age was 29.4 years (standard deviation (SD) 3.4 years) at childbirth. Mean prepregnancy BMI was 21.3 kg/m^2^ (SD 3.1 kg/m^2^). The proportion of maternal prepregnancy overweight was 14.0%, obesity 3.8%, and underweight 16.5%. During pregnancy, 48.7% of mothers had excessive GWG, and 15.1% had inadequate GWG. The incidence of GDM was 12.5%. Among the infants, 12.4% were LGA, and 4.0% were SGA. Gestational age ranged from 37–41 weeks.

### 3.1. Prenatal Factors and Birthweight for Gestational Age Status (AGA, LGA, SGA)

As shown in Table 1, the rate of OWO among mothers of LGA infants was higher than that among mothers of AGA infants, which was in turn higher than that among mothers of SGA infants.

The rate of excessive GWG was higher for mothers of infants who were LGA, and the rate of inadequate GWG was higher among mothers of infants who were SGA than AGA. In the LGA group, the proportion of mothers with OWO was 31%, and excessive GWG was 70%, both of which were higher than for mothers of infants in the other two groups (AGA and SGA). In contrast, in the SGA group, the proportion of maternal underweight was 18.8% and inadequate GWG was 40.6%, much higher than for mothers of infants in the other two groups (LGA and AGA). The proportion of C-section deliveries was higher among mothers of children who were LGA (88%) than for mothers of children who were AGA and SGA. The proportion of maternal GDM did not differ across the three infant growth trajectory groups (LGA, AGA, and SGA).

### 3.2. Longitudinal Data Analysis of the Age 0–2 Years Growth Trajectory in Term-Born AGA, LGA, and SGA Infants

#### 3.2.1. Growth Trajectories

Table 2 shows the mean growth slope with increasing age among AGA infants, compared to growth velocity differences in length, weight, BMI, and ZWFL in LGA and SGA infants, respectively.

As for length growth trajectories, the average growth rate was 3.00 (95% CI 2.97, 3.02) cm per month at age 0–6 months and 1.07 (95% CI 1.06, 1.08) cm/month at age 7–25 months in AGA newborns, with adjustment for covariates. Compared to AGA infants, the length growth rate was 0.1 cm/month slower for LGA infants at age 0–6 months, and 0.09 cm/month slower for SGA infants at age 7–25 months, with adjustment with covariates (Table 2).

**Figure 1:**
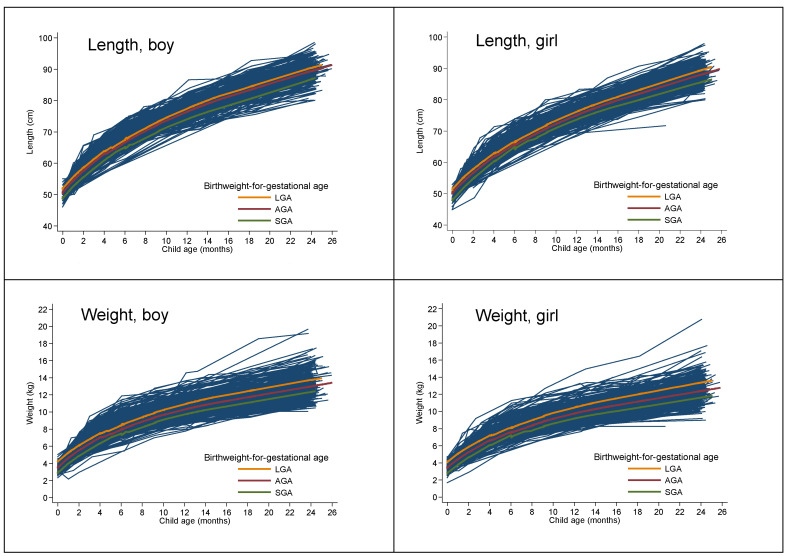

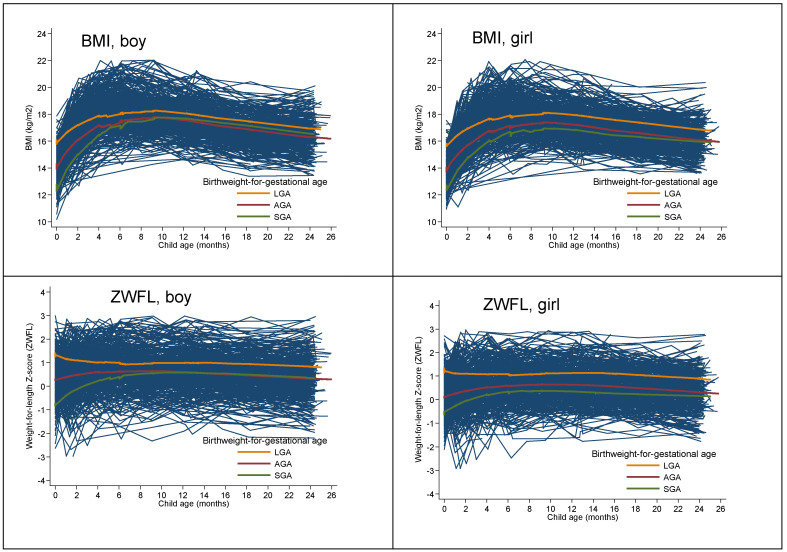
Spaghetti plot of 6340 longitudinal anthropometric adiposity measures from age 0–25 months and LOWESS regression plot by AGA, SGA, and LGA status in 806 term-born children (418 boys and 388 girls). AGA: appropriate for gestational age; SGA: small for gestational age; LGA: large for gestational age.

As for ZWFL, relative to the growth slope in AGA infants, the trajectory of ZWFL was 0.11 units/month higher (95% CI 0.04, 0.19) in SGA infants, and 0.12 units/month (95% CI −0.15, −0.08), which was lower for LGA infants at age 0–6 months, however, there was no difference from age 7–25 months (Table 2, Figure 1). Similar results were observed for BMI growth velocity (Table 2, Figure 1).

#### 3.2.2. Child Length, Weight, BMI, and ZWFL

Table 3 presents the overall mean difference in infant length, weight, BMI, and ZWFL in LGA and SGA versus AGA infants, at ages 0–6 months and 7–25 months, respectively.

As shown in Figure 1 and Table 3, overall, compared with AGA infants, SGA infants had consistently shorter length/length for age z-scores, and lower weight/weight for age z scores/ZWFL; while, in contrast, LGA infants had consistently higher values for all these anthropometric measures. For example, compared to AGA infants, SGA infants had consistently shorter lengths at ages 0–6 months (−1.81 cm, 95%CI: −2.36 to −1.26 cm), and at ages ≥7 months (−1.93 cm, 95% CI: −2.88 to −0.99), and lower weight at age 0–6 months (−0.63 kg), and at age ≥7 months (−0.65 kg). In contrast, LGA infants had consistently higher weights and slightly longer lengths, during the first two years of life (Table 3). 

The magnitudes of the differences were larger at age 0–6 months and became smaller at age 7–25 months for ZWFL, BMI, and weight for age z-scores for LGA versus AGA, as well as for SGA versus AGA infants. For instance, the differences between SGA and AGA infants became smaller for ZWFL (−0.51 versus −0.23), BMI (−0.98 versus −0.22 kg/m^2^), and weight for age z-score (−0.99 versus −0.52 unit), at age 0-6 months versus ages > 7 months, with adjustment for child age, child age^2^, maternal passive smoking during pregnancy, mode of delivery, infant sex, feeding type at age 0–6 months, and child passive smoking (Table 3). Similarly, the differences between LGA and AGA infants became smaller for ZWFL, BMI, and weight for age z-score at ages > 7 months compared to age 0–6 months.

### 3.3. AGA, LGA, and SGA Status and Blood Pressure at Age 2 Years

As shown in Table 4, being born SGA was associated with a 6.4 (95% CI 0.4–12.4) mmHg higher SBP while being born LGA was associated with a 2.9 (95% CI −5.2, −0.5) mmHg lower DBP in boys at age two years. No association was observed in girls.

## 4. Discussion

This prospective cohort study examined the overall contrasts in growth trajectories for infants born LGA, AGA, and SGA in the first two years of life. We found that LGA infants had consistently longer lengths and higher weights and ZWFL, while SGA infants had lower values for length and all the adiposity measures from age 0–2 years, relative to AGA infants. As for the growth trajectories, relative to AGA growth velocity, the trajectory of the ZWFL slope was greater for SGA infants and lower for LGA infants during the first six months. The differences in all the adiposity measures (weight for age z-score, ZWFL, and BMI) between SGA and AGA infants became smaller as they passed beyond age > 7 months. Being born SGA was associated with higher SBP, and being born LGA was associated with lower DBP in boys at age two years, however, these associations were not observed in girls.

In this study, all infants were term born, and intrauterine growth retardation (IUGR) restriction may have occurred for SGA infants. A previous study reported that the majority (86%) of children born full term and SGA eventually reached a “normal” length (i.e., above −2SD) during the first 6–12 months [23]. This study also indicated that SGA infants had continuously shorter recumbent lengths and were smaller over the first 24 months of life, suggesting that SGA is associated with restricted linear growth.

While SGA infants may exhibit catch-up growth, there is currently a lack of catch-up growth classifications for assessing SGA infants [24]. By using a longitudinal data analysis strategy and random effect models, this study found that increases in weight for age z-score, ZWFL and BMI at age 0–6 months were faster in SGA infants than in AGA infants. Poor fetal growth as a result of being SGA might program these infants to develop a smaller proportion of lean mass and a larger amount of fat mass [25]. Existing studies showed that SGA and overweight children were more insulin resistant and at increased risk of metabolic syndrome than AGA children, which might be due to adverse fetal programming and obesity [26].

This study delineated LGA and SGA growth profiles [27]. LGA infants tended to remain taller and heavier [27], while SGA infants remained shorter and lighter compared to AGA infants [28]. A recent study also reported that higher birthweight was associated with higher BMI at ages four and seven years [29,30]. In the present study, LGA infants had consistently higher adiposity measures than the other two (SGA and AGA) groups at age 0–2 years. Birthweight is closely related to later BMI, much of which may reflect the early programming of lean mass as opposed to fat mass [25]. Exactly how higher birthweight programs higher adulthood fat mass remains less clear [25]. Future studies with advanced body composition measures are required to unravel this issue.

Early-life intervention to prevent childhood obesity should be a priority for public health and clinical practice. For the present study, the study question of whether there were differences between growth trajectories of LGA, SGA, and AGA term-born infants in their first two years, and between their blood pressure at two years, was driven by clinical need. Early interventions to prevent obesity should be adapted to the risk status of each individual [15]. Using the DOHaD approach, an ongoing childhood obesity-intervention trial, the Healthy Life Trajectories Initiative (HeLTI) project adapted the intervention to consider the mother’s and child’s risk for child OWO in each phase of care (preconception, prenatal, postnatal, infancy, and childhood). In this model, mothers and/or their children were identified as being ‘at increased risk’ for childhood OWO, and in cases where risk factors for childhood OWO were identified, the intensity of the intervention was increased [15]. Findings from the present study suggest that both LGA and SGA infants should be considered as being at risk for OWO, and thus mothers and families probably should receive additional health promotion/information on how to maintain a healthy lifestyle in childhood and beyond.

Lower birthweight has been found to be associated with a higher risk of ischaemic heart disease in adult men [13,14]. Interestingly, our study found that being born SGA was associated with higher SBP while being born LGA was associated with lower DBP in boys at age 2 years. Our findings appear to be biologically plausible. Intrauterine growth restriction can cause a reduction in nephron number which can lead to the pathogenesis of hypertension [31]. Few studies have examined BP at the age of two years. Previous studies have mostly examined BP in school-age children and adolescents (age range 7–18 years), and their findings were inconsistent [32,33,34]. A cross-sectional study reported no association between birthweight and BP in adolescents (12–18 years) [32]. However, birthweight was also found to be negatively related to SBP [33] or to have a U-shaped relationship with the risk of hypertension in children and adolescents [34].

In this study, we observed a sex difference in the association between SGA/LGA and BP at age two years. On average, boys tended to be heavier and larger than girls at birth. This may imply that the same cutoff points for girls and boys may not be appropriate in the evaluation of low/high birthweight and related cardio-metabolic outcomes [35]. Accordingly, high birthweights in newborn girls might represent higher extremes than in newborn boys. Few previous studies have examined sex-specific associations between birthweight and later life BP, and existing findings are contradictory [36,37]. In this study, we used sex-specific cutoff points to define SGA and LGA in term-born infants. However, the mechanism underlying the sex difference in these associations is still largely unknown. Further prospective studies with longer follow-up periods of children to older ages are needed.

The strengths of this study include its prospective cohort design, high-quality clinical data on birth outcomes, and repeated anthropometric measures in young children. The study also had limitations. The SGA group was small in this study. In addition, anthropometric measurements were used in this study. More advanced assessments of body composition, including the use of stable isotope techniques, are needed to assess this population in future studies.

## 5. Conclusions

This prospective study demonstrated that infants born LGA had longer lengths and higher adiposity measures at age two years, and infants born SGA had a faster velocity in the trajectory of adiposity measures at age 0–6 months. SGA was associated with higher SBP, and LGA with lower DBP at age two years in boys only. Our study findings suggest that both LGA and SGA infants can be at increased risk of future obesity and cardiovascular diseases and need additional attention and education focused on individualized early prevention. The first 1000 days of life (from conception to age two years) is an important time period in which to address all forms of malnutrition and avoid the risk of ill health in later life.

## Figures and Tables

**Table 1:** Maternal and child characteristics by birthweight for gestational age status (AGA, SGA, and LGA) in 806 term-born children.

	AGA	LGA	SGA	*p* Value
N	674	100	32	
Maternal and Infant Characteristics				
Maternal Age at Childbirth (years)	29.4 ± 3.5	29.5 ± 3.2	29.5 ± 3.2	0.94
Prepregnancy BMI (kg/m^2^)	21.2 ± 3.0	22.8 ± 3.7	20.6 ± 2.6	<0.0001
Maternal Education				
Junior high school or lower	13 (1.9)	3 (3.0)	1 (3.1)	0.92
High school	71 (10.5)	12 (12.0)	3 (9.4)	
College or above	589 (87.5)	85 (85.0)	28 (87.5)	
Maternal Passive Smoke During Pregnancy				
Yes	196 (29.2)	33 (34.0)	8 (25.0)	0.53
No	475 (70.8)	64 (66.0)	24 (75.0)	
Maternal Prepregnancy BMI (kg/m^2^) Categories				
<18.5	120 (17.8)	7 (7.0)	6 (18.8)	0.002
18.5–23.9	443 (65.8)	62 (62.0)	23 (71.9)	
24–27.9	89 (13.2)	22 (22.0)	2 (6.3)	
>28	21 (3.1)	9 (9.0)	1 (3.1)	
Gestational Weight Gain				
Adequate	256 (38.4)	25 (25.0)	8 (25.0)	<0.0001
Excessive	308 (46.2)	70 (70.0)	11 (34.4)	
Inadequate	103 (15.4)	5 (5.0)	13 (40.6)	
Maternal GDM, Yes	84 (12.5)	12 (12.0)	5 (15.6)	0.86
Mode of Delivery				
Vaginal delivery	185 (27.4)	12 (12.0)	9 (28.1)	0.004
C-Section	489 (72.6)	88 (88.0)	23 (71.9)	
Gestational age (weeks)	39.0 ± 1.0	39.1 ± 1.0	38.8 ± 1.0	0.16
Birthweight (g)	3360 ± 282	4145 ± 239	2617 ± 249	<0.0001
Infant Sex				
Boy	354 (52.5)	51 (51.0)	13 (40.6)	0.41
Girl	320 (47.5)	49 (49.0)	19 (59.4)	
Feeding Type (0–6 months)				
Formula feeding	73 (12.7)	9 (10.7)	6 (23.1)	0.12
Exclusive Breastfeeding	204 (35.5)	34 (40.5)	13 (50.0)	
Mixed breastfeeding	297 (51.7)	41 (48.8)	7 (26.9)	
Child Exposure to Passive Smoking				
No	317 (48.2)	48 (50.0)	16 (50.0)	
Yes	341 (51.8)	48 (50.0)	16 (50.0)	0.93
Child Anthropometric Measures at 2 years				
N	451	68	23	
Child Age, Months	23.8 ± 0.5	23.7 ± 0.3	23.7 ± 0.3	0.48
Length	88.8 ± 3.0	89.9 ± 2.9	86.0 ± 3.9	<0.0001
Weight	12.8 ± 1.5	13.5 ± 1.3	12.0 ± 1.6	<0.0001
BMI (kg/m^2^)	16.2 ± 1.4	16.7 ± 1.2	16.2 ± 1.4	0.01
Weight for Length z-score (ZWFL)	0.37 ± 0.96	0.80 ± 0.84	0.30 ± 0.94	0.002
BMI for Age z-score	0.37 ± 0.98	0.78 ± 0.86	0.40 ± 0.97	0.005
Length for Age z-score	0.58 ± 0.94	0.98 ± 0.93	−0.23 ± 1.24	<0.0001
Weight for Age z-score	0.60 ± 0.91	1.12 ± 0.83	0.13 ± 1.01	<0.0001
Sum of Skinfold Thickness (mm)	22.8 ± 5.1	23.5 ± 5.2	22.8 ± 4.7	0.54
Midupper Arm Circumference (MUAC) (cm)	15.8 ± 1.2	16.2 ± 1.2	15.7 ± 1.0	0.05

AGA: appropriate for gestational age; SGA: small for gestational age; LGA: large for gestational age. Data are presented as mean ± SD, and n (%); χ^2^ tests or Fisher’s exact test were used for categorical variables and ANOVA F-tests were used for continuous variables.

**Table 2:** Longitudinal data analysis of birthweight for gestational age status and growth trajectories for length, weight, BMI, and weight for length z-score by age from birth to 6 months and from 7 to ~25 months in 806 children.

Predictors	Age 0–6 Months (Sample Size: 3262 Measures)				Age ≥ 7 Months (Sample Size: 3087 Measures)			
	Model 1		Model 2		Model 1		Model 2	
	β (95% CI)	*p*	β (95% CI)	*p*	β (95% CI)	*p*	β (95% CI)	*p*
Birthweight-for-GA	**Child Length (cm)**							
AGA	ref.		ref.		ref.		ref.	
LGA	1.31 (1.07, 1.54)	<0.001	1.33 (1.09, 1.56)	<0.001	0.49 (−0.10, 1.07)	0.11	0.49 (−0.09, 1.06)	0.10
SGA	−2.14 (−2.70, −1.58)	<0.001	−2.00 (−2.53, −1.48)	<0.001	−0.69 (−1.74, 0.37)	0.20	−0.54 (−1.59, 0.51)	0.31
Child Age	3.00 (2.97, 3.03)	<0.001	3.00 (2.97, 3.02)	<0.001	1.07 (1.06, 1.08)	<0.001	1.07 (1.06, 1.08)	<0.001
Child age × AGA	0.00		0.00		0.00		0.00	
Child age × LGA	−0.10 (−0.18, −0.02)	0.01	−0.10 (−0.18, −0.02)	0.011	0.03 (−0.005, 0.06)	0.10	0.03 (−0.005, 0.06)	0.10
Child age × SGA	0.04 (−0.08, 0.17)	0.51	0.04 (−0.08, 0.17)	0.50	−0.09 (−0.15, −0.03)	0.005	−0.09 (−0.15, −0.03)	0.005
	**Weight (kg)**							
AGA	ref.		ref.		ref.		ref.	
LGA	0.66 (0.59, 0.73)	<0.001	0.66 (0.59, 0.74)	<0.001	0.23 (−0.05, 0.51)	0.10	0.24 (−0.04, 0.52)	0.09
SGA	−0.71 (−0.85, −0.57)	<0.001	−0.67 (−0.80, −0.53)	<0.001	−0.46 (−0.88, −0.04)	0.03	−0.40 (−0.81, 0.009)	0.06
Child Age	0.84 (0.83, 0.85)	<0.001	0.84 (0.83, 0.85)	<0.001	0.22 (0.21, 0.22)	<0.001	0.22 (0.21, 0.22)	<0.001
Child age × AGA	0.00		0.00		0.00		0.00	
Child age × LGA	−0.04 (−0.07, −0.008)	0.01	−0.04 (−0.07, −0.008)	0.01	0.03 (0.01, 0.04)	<0.001	0.03 (0.01, 0.04)	<0.001
Child age × SGA	0.01 (−0.04, 0.05)	0.86	0.01 (−0.04, 0.05)	0.84	−0.02 (−0.05, 0.02)	0.31	−0.02 (−0.05, 0.02)	0.31
	**BMI (kg/m^2^)**							
AGA	ref.		ref.		ref.		ref.	
LGA	1.71 (1.52, 1.90)	<0.001	1.71 (1.52, 1.90)	<0.001	0.42 (−0.02, 0.85)	0.06	0.42 (−0.01, 0.86)	0.06
SGA	−1.59 (−1.96, −1.23)	<0.001	−1.56 (−1.93, −1.19)	<0.001	−0.67 (−1.32, −0.01)	0.05	−0.63 (−1.27, 0.007)	0.05
Child Age	0.74 (0.72, 0.76)	<0.001	0.74 (0.72, 0.76)	<0.001	−0.11 (−0.11, −0.10)	<0.001	−0.11 (−0.11, −0.10)	<0.001
Child age × AGA	0.00		0.00		0.00		0.00	
Child age × LGA	−0.23 (−0.28, −0.18)	<0.001	−0.23 (−0.28, −0.18)	<0.001	0.009 (−0.009, 0.03)	0.33	0.01 (−0.009, 0.03)	0.32
Child age × SGA	0.18 (0.09, 0.28)	<0.001	0.19 (0.09, 0.28)	<0.001	0.03 (−0.01, 0.06)	0.19	0.03 (−0.01, 0.06)	0.19
	**Weight for length z-score**							
AGA	ref.		ref.		ref.		ref.	
LGA	0.95 (0.81, 1.08)	<0.001	0.94 (0.81, 1.08)	<0.001	0.28 (0.01, 0.54)	0.04	0.28 (0.008, 0.54)	0.04
SGA	−0.84 (−1.14, −0.54)	<0.001	−0.85 (−1.14, −0.54)	<0.001	−0.40 (−0.80, 0.003)	0.05	−0.40 (−0.80, −0.005)	0.05
Child age (months)	0.10 (0.08, 0.11)	<0.001	0.10 (0.08, 0.11)	<0.001	−0.02 (−0.03, −0.02)	<0.001	−0.02 (−0.03, −0.02)	<0.001
Child age × AGA	0.00		0.00		0.00		0.00	
Child age × LGA	−0.12 (−0.15, −0.08)	<0.001	−0.12 (−0.15, −0.08)	<0.001	0.009 (−0.002, 0.02)	0.12	0.009 (−0.002, 0.02)	0.12
Child age × SGA	0.11 (0.04, 0.19)	0.002	0.11 (0.04, 0.19)	0.002	0.01 (−0.01, 0.04)	0.37	0.01 (−0.01, 0.04)	0.38

Model 1: y = β0 + β1 * LGA + β2 * SGA + β3 * child age + β4 * child age * LGA + β5 * child age * SGA; Model 2: y = β0 + β1 * LGA + β2 * SGA + β3 * child age + β4 * child age * LGA + β5 * child age * SGA + covariates. Covariates included in the model were maternal passive smoking during pregnancy (yes/no), mode of delivery, infant sex, feeding type at age 0–6 months, and child passive smoking (yes/no).

**Table 3:** Associations between birthweight-for-gestational age status (AGA, LGA, and SGA) and infant length, weight, and weight-for-length z-score from birth to age 25 months in 806 children.

Predictors	0–6 months				≥7 months			
	Crude Model		Adjusted Model		Crude Model		Adjusted Model	
	β (95% CI)	*p*	β (95% CI)	*p*	β (95% CI)	*p*	β (95% CI)	*p*
Birthweight for GA	**Child Length (cm)**							
AGA	ref.		ref.		ref.		ref.	
LGA	0.32 (−0.69, 1.33)	0.54	1.06 (0.77, 1.35)	<0.0001	1.16 (0.57, 1.74)	0.0001	0.88 (0.38, 1.39)	0.0006
SGA	−2.69 (−3.81, −1.57)	<0.0001	−1.81 (−2.36, −1.26)	<0.0001	−2.26 (−3.40, −1.12)	0.0001	−1.93 (−2.88, −0.99)	<0.0001
	**Weight (kg)**							
AGA	ref.		ref.		ref.		ref.	
LGA	0.51 (0.36, 0.66)	<0.0001	0.56 (0.45, 0.67)	<0.0001	0.67 (0.43, 0.92)	<0.0001	0.63 (0.39, 0.86)	<0.0001
SGA	−0.7 (−0.94, −0.47)	<0.0001	−0.63 (−0.84, −0.42)	<0.0001	−0.73 (−1.08, −0.38)	<0.0001	−0.65 (−0.97, −0.32)	<0.0001
	**BMI (kg/m^2^)**							
AGA	ref.		ref.		ref.		ref.	
LGA	1.05 (0.83, 1.27)	<0.0001	1.11 (0.90, 1.31)	<0.0001	0.56 (0.31, 0.81)	<0.0001	0.57 (0.32, 0.82)	<0.0001
SGA	−0.98 (−1.37, −0.59)	<0.0001	−0.98 (−1.38, −0.58)	<0.0001	−0.24 (−0.55, 0.06)	0.12	−0.22 (−0.51, 0.06)	0.13
	**Length for age z-score**							
AGA	ref.		ref.		ref.		ref.	
LGA	0.52 (0.38, 0.66)	<0.0001	0.52 (0.39, 0.66)	<0.0001	0.34 (0.15, 0.53)	0.0004	0.33 (0.14, 0.52)	0.0008
SGA	−0.86 (−1.13, −0.60)	<0.0001	−0.89 (−1.15, −0.63)	<0.0001	−0.70 (−1.07, −0.34)	0.0002	−0.71 (−1.06, −0.36)	<0.0001
	**Weight for age z-score**							
AGA	ref.		ref.		ref.		ref.	
LGA	0.82 (0.70, 0.94)	<0.0001	0.82 (0.70,0.95)	<0.0001	0.47 (0.29,0.64)	<0.0001	0.46 (0.28,0.64)	<0.0001
SGA	−0.96 (−1.27, −0.66)	<0.0001	−0.99 (−1.29,−0.68)	<0.0001	−0.51 (−0.77,−0.25)	0.0001	−0.52 (−0.77,−0.27)	<0.0001
	**Weight for length z-score (ZWFL)**							
AGA	ref.		ref.		ref.		ref.	
LGA	0.62 (0.49, 0.75)	<0.0001	0.62 (0.49, 0.75)	<0.0001	0.42 (0.25, 0.59)	<0.0001	0.42 (0.25, 0.59)	<0.0001
SGA	−0.5 (−0.77, −0.23)	0.0003	−0.51 (−0.79, −0.24)	0.0003	−0.22 (−0.42, −0.02)	0.03	−0.23 (−0.43, −0.03)	0.02

The adjusted models included child age, child age^2^, maternal passive smoking during pregnancy (yes/no), mode of delivery, infant sex, feeding type (exclusive breastfeeding, mixed feeding, and formula) at age 0–6 months, and child passive smoking (yes/no).

**Table 4:** The associations between birthweight for gestational age status (LGA, AGA, and SGA) and blood pressure in children at age 2 years.

Birthweight for Gestational Age	N	SBP (mmHg)			DBP (mmHg)		
		Mean ± SD	Β (95% CI)	*p* Value	Mean ± SD	Β (95% CI)	*p* Value
		**Boys**					
AGA	244	93.0 ± 8.1			61.5 ± 6.2		
SGA	8	98.5 ± 11.0	6.4 (0.4, 12.4)	0.04	64.0 ± 6.8	2.9 (−1.9, 7.7)	0.23
LGA	36	92.9 ± 8.1	−1.5 (−4.4, 1.5)	0.33	59.5 ± 6.1	−2.9 (−5.2, −0.5)	0.02
		**Girls**					
AGA	219	91.6 ± 7.2			61.1 ± 5.4		
SGA	15	90.5 ± 8.8	−0.3 (−4.4, 3.8)	0.88	61.8 ± 9.4	1.1 (−2.1, 4.3)	0.49
LGA	36	94.3 ± 9.1	0.8 (−2.0,3.7)	0.56	62.1 ± 5.9	−0.2 (−2.4, 2.0)	0.87

All models were adjusted for child age and weight for length z-score at age 2 years.

## Data Availability

Data is not readily available because access to the deidentified participant research data must be approved by the research ethics board on a case-by-case basis. Please contact the corresponding author (ouyangfengxiu@xinhuamed.com.cn) for assistance in data access request.
